# Development of a Tightly Controlled Off Switch for *Saccharomyces cerevisiae* Regulated by Camphor, a Low-Cost Natural Product

**DOI:** 10.1534/g3.114.012765

**Published:** 2015-07-22

**Authors:** Shigehito Ikushima, Yu Zhao, Jef D. Boeke

**Affiliations:** * High Throughput Biology Center and Department of Molecular Biology and Genetics, Johns Hopkins University School of Medicine, Baltimore, Maryland 21205; † Central Laboratories for Key Technologies, KIRIN Company Limited, Yokohama, Kanagawa 236-0004, Japan; ‡ Institute for Systems Genetics and Department of Biochemistry and Molecular Pharmacology, New York University Langone Medical Center, New York, New York 10011

**Keywords:** CamR, *Pseudomonas putida*, TetR homolog, Tet system

## Abstract

Here we describe the engineering of a distant homolog of the Tet repressor, CamR, isolated from *Pseudomonas putida*, that is regulated by camphor, a very inexpensive small molecule (at micromolar concentrations) for use in *Saccharomyces cerevisiae*. The repressor was engineered by expression from a constitutive yeast promoter, fusion to a viral activator protein cassette, and codon optimization. A suitable promoter responsive to the CamR fusion protein was engineered by embedding a *P. putida* operator binding sequence within an upstream activating sequence (UAS)-less *CYC1* promoter from *S. cerevisiae*. The switch, named the Camphor-Off switch, activates expression of a reporter gene in camphor-free media and represses it with micromolar concentrations of camphor.

The budding yeast *Saccharomyces cerevisiae* is one of the most important organisms in both applied and fundamental biology. Numerous studies of regulated protein expression have been performed, and currently there are two types of regulated promoters available in the yeast (Maya *et al.* 2008). The first type is an innate yeast promoter, typified by the very well-known *GAL* promoters, the expression of which is repressed by glucose and activated by galactose . The *GAL1* promoter is used in many cases because its induction ratio is very high, but the high concentration of galactose required in the system as well as the slow growth and altered metabolism of yeast grown on galactose can be problematic for many applications. Apart from the *GAL* promoters (Johnston and Davis 1984), repressive promoters from the *MET3* gene (negatively regulated by methionine) (Mao *et al.* 2002) and *PHO5* (negatively regulated by inorganic phosphate) (Kaneko *et al.* 1985) are available. However, the use of these promoters has multiple potentially undesirable effects on metabolism and host gene transcription and, importantly, can also lead to a slow growth rate and/or a high cost incompatible with biotechnological applications, making them suboptimal.

The other type of promoters consists of synthetic functional units derived from other organisms, such bacteria and viruses. One of the most studied switches, called the Tet system, has been applied to regulate expression in yeast (Gari *et al.* 1997). In that system, a transcription factor, the TetR protein from *Escherichia coli*, can bind to its operator sequence depending on the presence or absence of tetracycline or derivative compounds such as anhydrotetracycline or doxycycline. However, use of antibiotics hinders large-volume fermentation in industry because of their expense. Thus, a low-cost alternate system is desirable because it could become a method of choice for regulated protein and pathway expression for large-scale yeast fermentation, for example, at a scale of millions of liters. In addition, it is obvious that an increase in the number of available ligand-activated switches provides more options for both fundamental and applied studies.

Previous articles have described an autoregulated camphor oxidation operon in the 240-kb plasmid PpG1 from *Pseudomonas putida* (Fujita *et al.* 1993; Aramaki *et al.* 1993). Expression of this operon is induced by the presence of camphor, because a TetR-homolog transcription factor, CamR, dissociates from the bound operator when bound to the camphor ligand. Notably, camphor is very inexpensive and widely used in human daily life and therapeutically. These facts attracted us to this camphor system as a potential gene regulatory system for the yeast *S. cerevisiae*. Here we describe the engineering of the system and the minimal impact of camphor on the growth of yeast.

## Materials and Methods

### Media

Yeast strains were cultured in YPD medium or SD-based media supplemented with appropriate amino acids; fully supplemented medium containing all amino acids plus uracil and adenine is referred to as SC. SC–Ade is SC lacking adenine. D-Camphor was purchased from Sigma-Aldrich (St. Louis, MO), and 5-fluoroorotic acid (5-FOA) was from US Biological (Massachusetts, MA). Doxycycline (Dox) was obtained from Clontech laboratories (Mountain View, CA). *Escherichia coli* was grown in Luria Broth (LB) media. To select strains with drug-resistant genes, carbenicillin (Sigma-Aldrich), kanamycin (Sigma-Aldrich), or zeocin (Life Technologies, Carlsbad, CA) were used at final concentrations of 75 µg/ml, 50 µg/ml, and 25 µg/ml, respectively. Agar was added to 2% for preparing solid media.

### Plasmids

The plasmids were constructed with protocols described previously (Mitchell *et al.* 2013). In this study, the TOP10 strain of *E. coli* [F– *mcr*AΔ(*mrr-hsd*RMS-*mcr*BC) Φ80*lac*ZΔM15 Δ*lac*X74 *rec*A1 *ara*D139 Δ(*araleu*) 7697 *gal*U*gal*K*rps*L (StrR) *end*A1 *nup*G] was used for the construction and amplification of plasmids.

**Figure 1:**
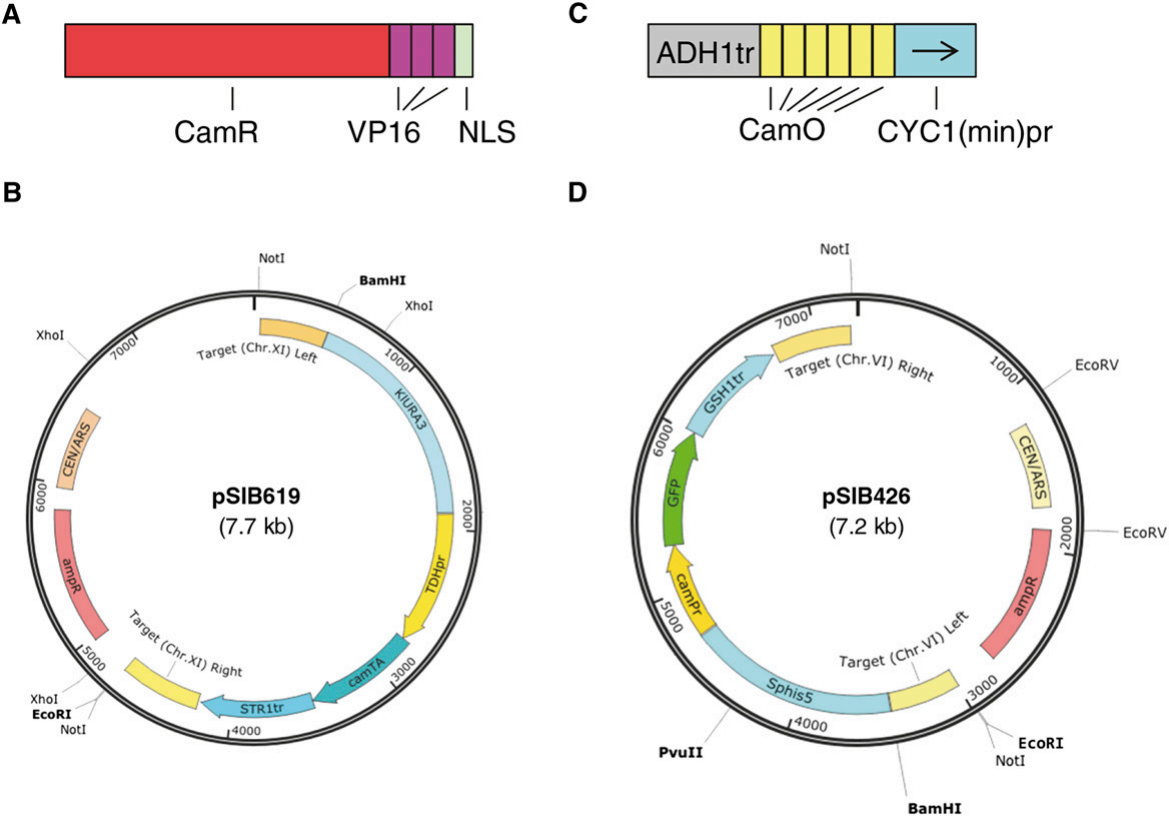
Diagram of Camphor-Off switch components in *S. cerevisiae*. (A) CamR-based transcriptional regulator (cam-TA) consisting of CamR, a trimeric repeat of VP16 transcription activation domain, and SV40 nuclear localization signal (NLS). (B) Plasmid pSIB619 for expressing cam-TA. *Not*I digestion releases an integration cassette ready to integrate into a neutral site in chromosome XI. (C) Architecture of a promoter designed to regulate the expression of genes of interest. The promoter camPr consists of the *ADH1* transcriptional terminator, six repeats of the CamR operator (CamO), and the *CYC1* minimal promoter, *CYC1*pr(min). (D) Plasmid pSIB426, harboring a GFP reporter. A similar plasmid, pSIB791, contains the *ADE2* gene in place of the *gfp* reporter in pSIB426.

Plasmids pSIB233, pSIB619, pSIB426, and pSIB791 are “convertible” vectors, meaning they can be used either as a CEN (single copy, episomal) plasmid directly or, upon cutting with *Not*I, for integration at chromosomes XI, IV, and VI at sites in large gene-free regions, and are empirically determined to be nondeleterious to yeast growth. pSIB619 (Figure 1C) was constructed by cloning a gene expression cassette that comprised the *TDH1* promoter, cam-TA (Supporting Information, Figure S1A), and the *STR1* terminator into pSIB233. In addition, the GFP reporter plasmid pSIB426 (Figure 1D) was constructed by tethering camPr (Figure S1B), the *gfp* CDS (coding sequence), and the *GSH1* terminator. The *ADE2* CDS was cloned in place of the *gfp* gene in pSIB426 to build an *ADE2* reporter plasmid pSIB791.

Plasmids pSIB872 and pSIB527 are integration vectors designed to integrate into chromosomes IV (*HO* gene) and VI with different yeast selection markers (Figure 6, A and B). pSIB872 was constructed by cloning two transcriptional units (TUs). One consisted of *TDH1*pr, the cam-TA CDS, and *STR1*tr; the other comprised the camPr, the mCherry CDS, and the *SOL3* terminator. In addition, the Tet-Off switch plasmid pSIB527 was built by cloning the following two TUs. One had human *CMV* promoter from human cytomegalovirus, codon usage optimized tTA (a TetR-VP16 fusion)(5), and the *STR1*tr. The other carries the *tet* promoter (tetPr), *gfp*, and *GSH1*tr. The tetPr itself consists of three subparts, the *ADH1*tr, *tet* operator, and *CYC1* minimal promoter.

### Yeast strains

Yeast strains are listed in Table 1. Strain BY-ade2∆ was constructed as follows: BY4741 was transformed with *Bam*HI-digested p∆ADE2 (Aparicio *et al.* 1991) followed by the selection of Ura^+^ colonies, and then Ura^−^ Ade^−^ derivatives were subsequently identified by screening for Ade^−^ colonies after selection on SC + 100-µg/ml 5-FOA medium. The other strains were constructed by integrating the expression cassettes that were prepared by digesting the aforementioned pSIB series of plasmids with *Not*I and selecting for the appropriate marker. Yeast cells were cultured at 30°.

**Table 1:** Yeast strains used in this study

Strain	Alternate Name	Genotype	Source
BY4741		MATa *his3Δ1 leu2Δ0 met15Δ0 ura3Δ0*	Lab stock
camG	SIY504	BY4741 targetChVI*^a^*::pSIB426 (*Sphis5*, camPr-*gfp*)	This study
camG-TA	SIY733	BY4741 targetChVI::pSIB426 (*Sphis5*, camPr-*gfp*)	This study
		*ykl162c*::pSIB619 (*KlURA3*, cam-TA)	
BY-ade2Δ	SIY390	BY4741 *ade2Δ*::*hisG* (derived from p∆ADE2)	This study
camA-TA	SIY794	BY-ade2Δ targetChVI::pSIB791 (*Sphis5*, camPr-*ADE2*) *ykl162c*::pSIB619 (*KlURA3*, cam-TA)	This study
camA-EmV	SIY795	BY-ade2Δ targetChVI::pSIB791 (*Sphis5*, camPr-*ADE2*) *ykl162c*::pSIB233 (*KlURA3*)	This study
BY4742		*MATalpha his3Δ1 leu2Δ0 lys2Δ0 ura3Δ0*	Lab stock
TetOffG	SIY555	BY4742 targetChVI::pSIB527 (*Sphis5*, tetPr-*gfp*, tTA)	This study
BY-TeCaOFF	SIY797	BY4742 *ho*::pSIB872 (*KlURA3*, camPr-*rfp*, cam-TA) targetChVI::pSIB527 (*Sphis5*, tetPr-*gfp*, tTA)	This study
YAS38		BY4741 bar1:: pFus1-yEGFP, pFUS1-tdTomato - LEU2	Lab stock
camL	YZY009	BY4741 targetChII*^b^*:: *Sphis5*, camPr	This study
WT-TA	SIY706	BY4741 *ykl162c*::pSIB619 (*KlURA3*, cam-TA)	This study
CamL - TA	YZY011	BY4741 targetChII*^b^*:: *Sphis5*, camPr	This study
		*ykl162c*::pSIB619 (*KlURA3*, cam-TA)	

a TargetChVI: an intergenic region between *GAT1*/*YFL021W* and *PAU5*/*YFL020C*.

b TargetChII: the intergenic region between TKL2/YBR117C and the open reading frame of LYS2/YBR115C.

### Flow cytometry

Cellular fluorescence from GFP was determined using the LSRII flow cytometer (Becton Dickinson, Franklin Lakes, NJ) equipped with 488-nm argon ion laser (blue laser) and a 530/30 filter. All samples were suspended in sterile water, and 10,000 cells were analyzed per sample. Data acquisition and analysis were performed using FACSDiva software (Becton Dickinson) and FlowJo (Tree Star, Ashland, OR).

### Growth tests

Yeast growth was monitored using an Eon Microplate Spectrophotometer with Gen5 software (BioTek, Winooski, VT), and 96 Well Clear Flat Bottom plates (Corning, NY) were used to culture the cells. Fresh cells were suspended in the required medium, and then 200 µl of the samples were filled in each well. Typically, the initial value of A_600_ was 0.20 to 0.25, and the absorbance was measured at 600 nm every 10 min. Doubling time was determined from cells that were in logarithmic phase.

### Microscopy

Cells were viewed with an Axioskop-2 microscope (Carl Zeiss, Oberkochen, Germany) equipped with an X-cite120 light source (ExFo, Ontario, Canada) and a fluorescence filter set. A 100× objective and AxioVision software were used to capture fluorescence and differential interference contrast (DIC) images.

### Immunoblotting

Yeast cells were picked into 5 ml YPD media from single colonies. After overnight growth, 5 µl of culture was added to 5 ml YPD or SC medium with or without 25 µM camphor. Cells were incubated 24 hr at 30°; 200 µl yeast cells were collected and washed with water and then incubated in 100 µl of 0.2 M NaOH for 5 min at room temperature. The cells were pelleted and resuspended in 59 µl SDS sample buffer (0.06 M Tris-HCL pH 6.8, 5% glycerol, 2% SDS, 4% beta-mercaptoethanol, 0.0025% bromophenol blue); 4–20% Tris-Glycine SDS page gels (Life Technologies, Carlsbad, CA) were run at 80 V for 10 min followed by 120 V for 2 hr. The Semi-Dry and Rapid Blotting System (Bio-Rad Laboratories, Inc, Hercules, CA) was used to transfer proteins to a PVDF membrane (Bio-Rad Laboratories, Inc, Hercules, CA). Anti-GFP antibody from mouse (Cat# 11814460001; Roche) and TUBB2A from rabbit (Cat# AV40177; Sigma-Aldrich) were mixed and used as the primary antibody. IRDye Goat anti-Mouse IgG (Cat# 926-32210; LI-COR Biosciences) and Goat anti-Rabbit IgG (Cat# 926-68071; LI-COR Biosciences) were used as secondary antibodies, respectively. The fluorescence signal was detected on an Odyssey CLx Imager from LI-COR (Lincoln, Nebraska).

### Data availability

Requests for all plasmids and strains and requests for more detailed supporting data are available upon request.

## Results and Discussion

### Construction of a CamR-based transcriptional regulator for *S. cerevisiae*:

We constructed a transcriptional regulator named cam-TA consisting of *Pseudomonas putida* CamR (GenBank BAA03510.1), three tandem repeats of a VP16 transcriptional activation domain derived from herpes simplex virus type 1 (Baron *et al.* 1997), and a nuclear localization signal (NLS) from SV40 (Kalderon *et al.* 1984) codon-optimized using GeneDesign (Richardson *et al.* 2006) (www.genedesign.org) for expression in yeast (Figure 1A). The glycolytic promoter *TDH1* was used to drive cam-TA expression (Figure 1B).

### Construction of a camphor responsive promoter: using GFP as a reporter

CamR, a component of cam-TA, binds to a specific sequence, 5′-CAGGCTCTATATCTGCGATATACTGAGCAT (Fujita *et al.* 1993). We assembled six repeats of the operator sequence (camO) separated by a junction sequence, 5′-CCCCC, used previously (Aramaki *et al.* 2011) between the alcohol dehydrogenase *ADH1* terminator and a *CYC1* (cytochrome c) promoter from which the endogenous UAS had been previously removed. The architecture, consisting of a terminator, an operator, and a UAS-less promoter, was analogous to a promoter used in the yeast Tet-system (Gari *et al.* 1997). This camO-containing promoter (camPr) was designed to regulate expression of genes of interest.

### Performance of the new switch: GFP as a reporter

**Figure 2:**
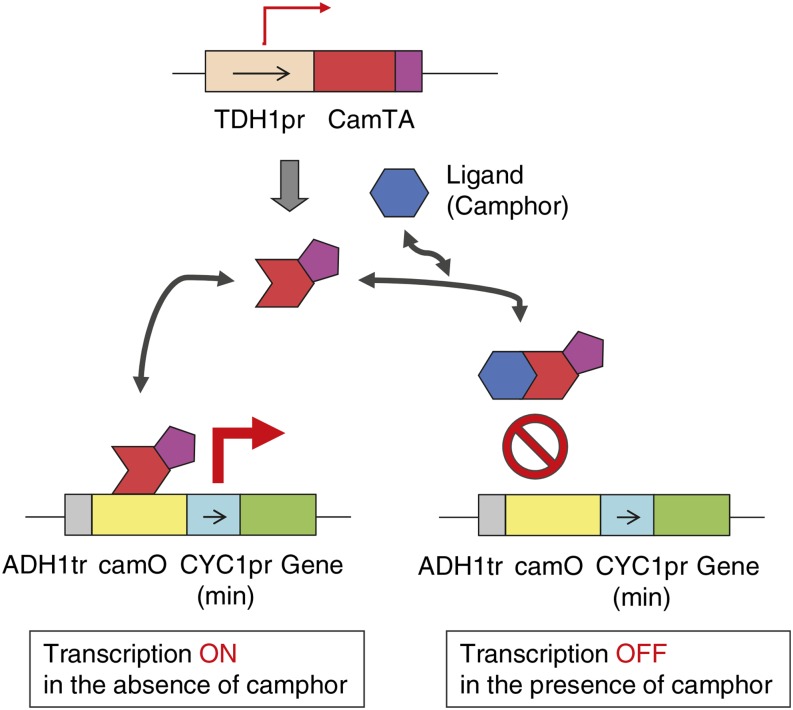
Schematic diagram of the Camphor-Off switch system.

We assessed the performance of the Camphor-Off system by combining the two components, cam-TA and camPr, described in the previous sections. In an effective system, we expected that a reporter gene under the control of camPr would express in the absence of camphor, a ligand of CamR, but that the expression of the reporter would be repressed by a certain amount of camphor (Figure 2). To evaluate the performance of the system, we used GFP as a reporter.

**Figure 3:**
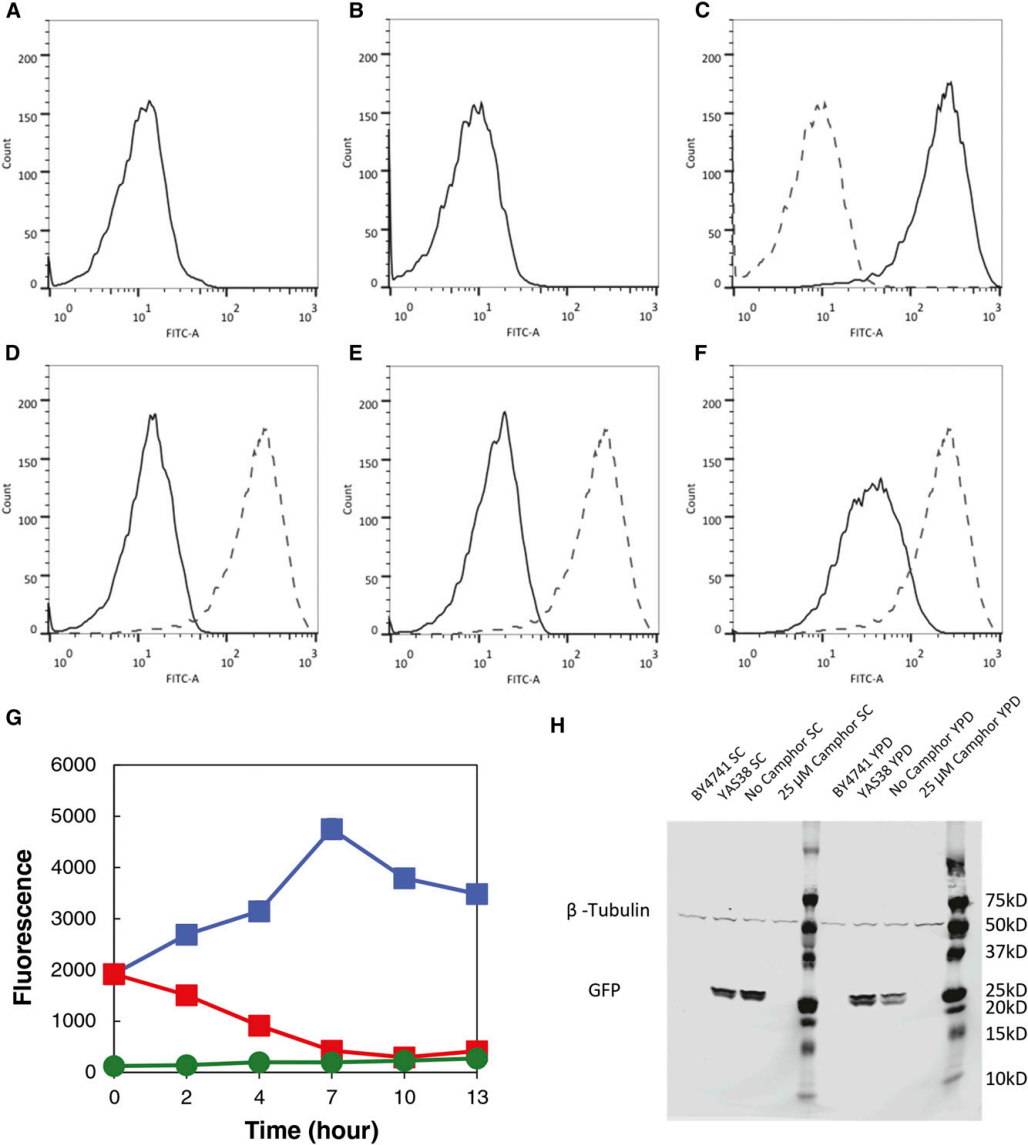
Characteristics of the Camphor-Off switch. (A–F) Cells were cultured in SC medium without camphor (A, B, and C) and with 100 µM (D), 25 µM (E), and 13 µM (F) of camphor. The strains were BY4741 (A), camG (B), and camG-TA (C–F). FITC-A shows the intensity of GFP fluorescence. (G) Time course of GFP fluorescence. The *y*-axis is equal to mean values of fluorescence out of 10,000 counts. Blue and red squares represent the camG-TA cells grown in the absence or presence of camphor (N = 5), respectively. Green circle shows BY4741 grown without camphor (N = 3). Error bars reflect SD. (H) Western Blot detection of GFP level in the camG-TA strain. BY4741 and YAS38 are used as the negative control and positive control, respectively.

The intensity of GFP did not vary between the host strain BY4741 and a control strain camG containing the camPr-*gfp* expression cassette but lacking the cam-TA construct (Table 1 and Figure 3, A and B). However, another strain, camG-TA, that was constructed by introducing the cam-TA-*gfp* expression cassette into the cam-TA strain produced significant amounts of GFP (Figure 3C). From this we conclude that cam-TA binds to camO and can activate the transcription of the reporter gene *gfp*. We then cultured the camG-TA strain in medium containing different camphor concentrations. The GFP signal drastically decreased in the presence of ≥25 µM of camphor (Figure 3, D–F), consistent with the camphor ligand dissociating cam-TA from camO. The lack of overlap between the two peaks (zero *vs.* 25 µM camphor) and their sharpness suggest that switch states are quite stable.

Subsequently, the kinetics of the system was investigated using the camG-TA strain. The intensity of GFP began high in the absence of camphor, whereas it went to almost the same level as that of BY4741 over a 7-hr period in the presence of camphor (Figure 3G). It was reasonable that the signal did not disappear immediately after the addition of camphor because native GFP has a half-life of approximately 7 hr in yeast. This is consistent with a relatively fast off switch.

To further confirm switch-off performance, we performed an immunoblot experiment to detect the GFP level controlled by camphor. In cells incubated for 24 hr with 25 µM camphor, no GFP protein is detected but a strong band appears for the cells incubated without camphor (Figure 3). Consistent results were obtained in both SC and YPD media. Thus, camphor efficiently switches off expression of GFP under the control of this camphor system.

### Camphor-Off system with an *ADE2* reporter

**Figure 4:**
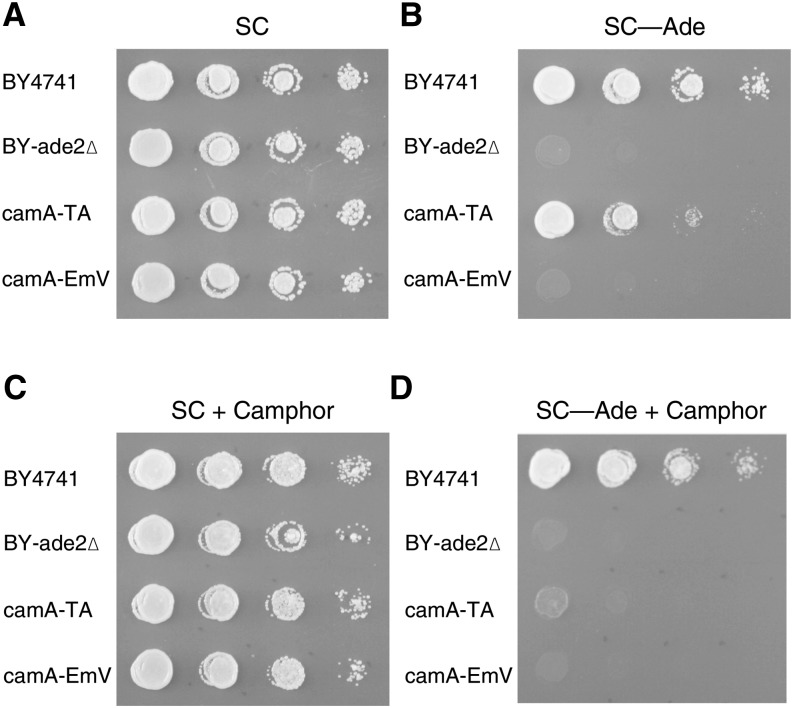
Camphor responsive *ADE2*-reporter. Cells were cultured for 1 d at 30°C in SC (A), SC–Ade (B), SC with 100 µM of camphor (C), and SC–Ade with 100 µM of camphor (D). The cells were diluted 10-fold across each row.

Another gene, *ADE2*, was used to further evaluate the Camphor-Off system. Specifically, we investigated availability of the system to complement the adenine-auxotrophy in a camphor-dependent manner. When cam-TA was not expressed in a strain that had camPr-controlled *ADE2* gene, the strain, named camA-EmV (camA Empty-vector), grew in SC medium but not in SC–Ade medium, irrespective of camphor addition (Figure 4). However, strain camA-TA, expressing the cam-TA protein, grew in camphor-free SC–Ade medium (Figure 4B). By contrast, the strain hardly proliferated at all on SC–Ade+Cam medium (containing camphor; Figure 4D). Similarly, no clone appeared in the SC–Ade+Cam but nearly 1000 clones occurred in camphor-free SC–Ade medium (Figure S1). The reversion frequency of this construct on SC–Ade is approximately 10^−3^. The behavior of the strain was consistent with the expectations of the design of the camphor system (Figure 2). The result showed that the new system constructed in this study was useful for tight regulation of two different genes in yeast.

### Growth effects of camphor treatment on yeast cells

**Figure 5:**
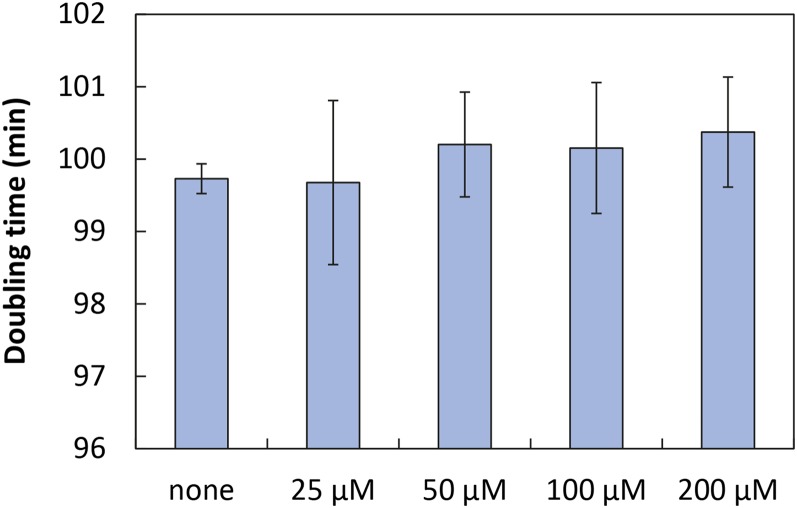
Doubling time of BY4741 strain in the presence of various concentrations of camphor. The values are means and SD calculated from eight experiments.

**Figure 6:**
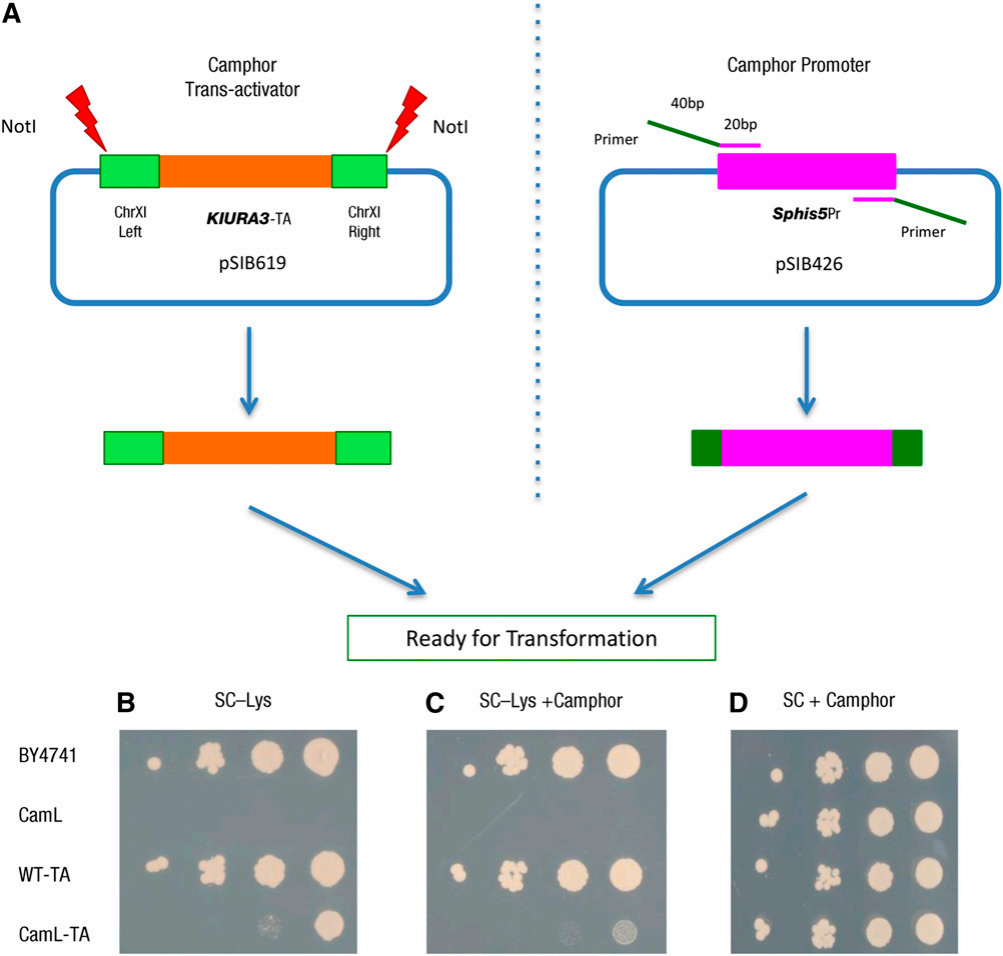
Workflow of one-step integration into any potential chromosome location. (A) After *Not*I digestion, the camphor *trans*-activator is ready to be integrated into Chr *XI* between *GAT1/YFL021W* and *PAU5/YFL020C*. The camphor promoter can be easily amplified by PCR with the primer containing the 40 bp homologous region (green) from the target chromosome location for the integration and 20 bp region (magenta) for annealing. Based on the one-step integration strategy, the *LYS2* gene was used to confirm the efficiency of shutoff. Cells were cultured for 1 d at 30° in SC–Lys (B), SC–Lys +100 µM camphor (C), and SC with 100 µM of camphor (D). The cells were diluted 10-fold in across each row.

The effective concentration for the Camphor-Off system was found to be 25 µM (Figure 3). Based on this, we investigated how yeast cells were affected by camphor by measuring the doubling time of BY4741 in camphor-containing medium. When BY4741 was cultivated in five different concentrations of camphor (none, 25 µM, 50 µM, 100 µM, and 200 µM), there was no significant difference between these conditions (Figure 5). Related to the experiment, a previous study claimed no mutagenic effect of camphor in the Ames test at concentrations as high as 500 mM (Gomes-Carneiro *et al.* 1998). Thus, exposure of yeast cells to camphor seemingly has little impact on yeast physiology (Figure 6).

### Camphor-Off system applied to an endogenous target gene

For prospective applications in the laboratory and industry, it would be useful if the Camphor-Off system could potentially be applied to any endogenous gene in *S. cerevisiae*. A versatile PCR-based integration strategy for the *GAL* promoter has been described (Longtine *et al.* 1998). Here, we designed an analogous one-step integration strategy for the Camphor-Off system, which can be applied to any potential chromosome location. For the camphor *trans*-activator, this module is ready for transformation and integration into the chromosome after *Not*I digestion. For the camphor promoter (tagged with the *Sphis5* marker), this module can be easily amplified with a pair of primers, which consist of a 40-bp targeting homology region for integration and a 20-bp annealing region to the end of the promoter cassette (Figure 6A). Then, two products are ready for co-transformation into the target strain. To test the efficiency, we used *LYS2* gene as the target. When the camphor *trans*-activator was not expressed, the CamL strain in which the *LYS2* is under the control of camphor promoter cannot grow on SC–Lys plate or SC–Lys + camphor plate. In contrast, if only the camphor *trans*-activator is integrated (WT-TA strain), then the *LYS2* behaves normally compared to the WT strain. The strain camL-TA with a functional cam-TA protein grew in a camphor-free SC–Lys plate (Figure 6B), but the strain grew very poorly on SC–Lys+Cam medium, indicating control by camphor (Figure 6C).

### Orthogonality: two different off switches in a single strain

**Figure 7:**
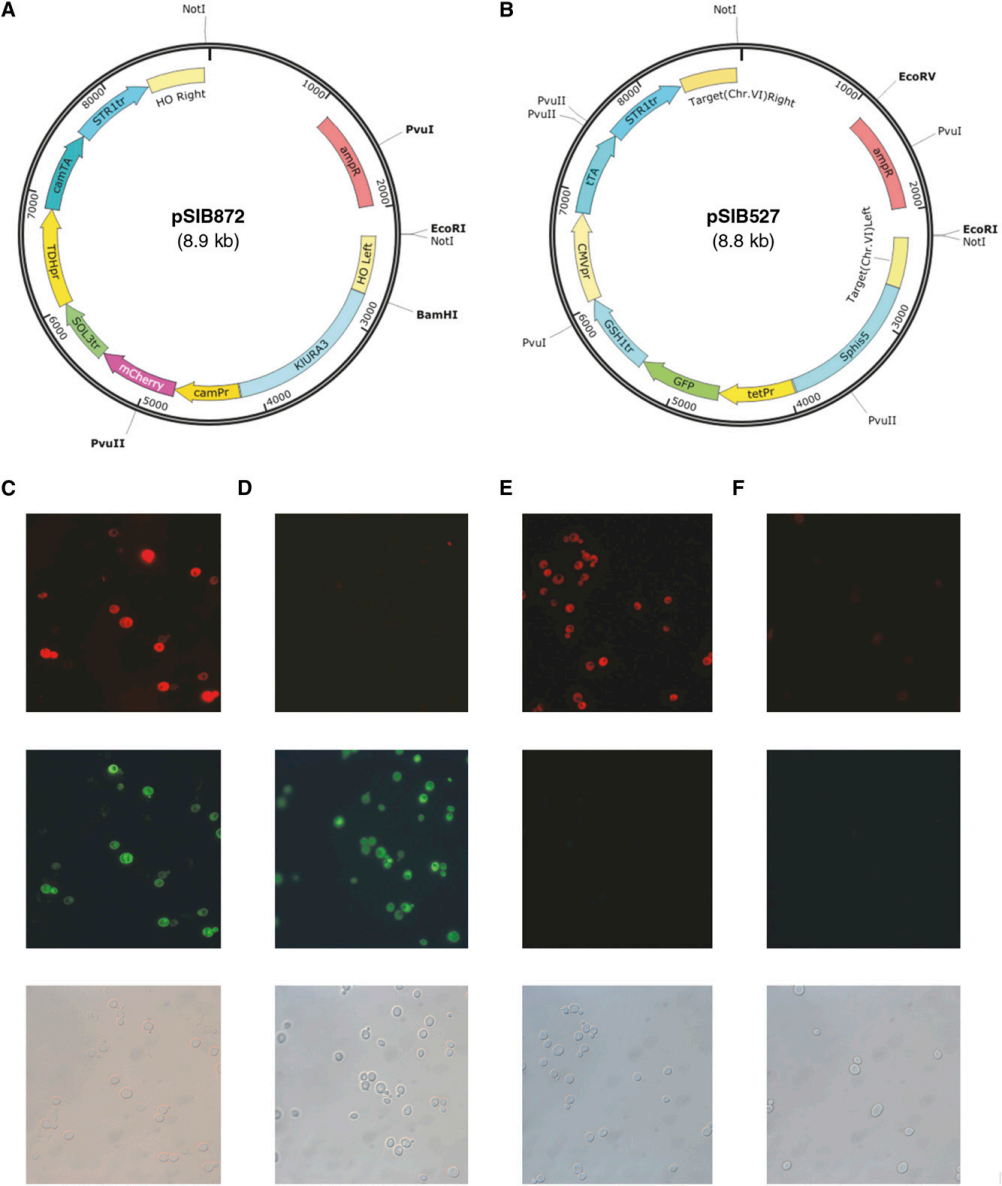
Independent regulation of fluorescent reporters, RFP and GFP, with Camphor-Off and Tet-Off systems. (A) pSIB872 carries a reporter of mCherry under control of the Camphor-Off system. (B) Plasmid pSIB527 carries *gfp*, the expression of which is regulated with the Tet-Off system. (C–F) Strain BY-TeCaOFF carries both pSIB872 and pSIB527. Cell images in SC medium (C), SC with camphor (D), SC with Dox (E), and SC with camphor and Dox. The top, middle, and bottom rows represent images of RFP, GFP, and DIC, respectively. Strain BY-TeCaOFF was grown for 1 d in SC medium with the indicated compounds. Camphor and Dox were supplemented at 25 µM each.

The experiments in this section were performed to examine whether the Camphor-Off system was orthogonal/compatible with the Tet-Off system in a yeast strain. First, we constructed two vectors: plasmid pSIB872 harbored the mCherry gene under control of the Camphor-Off switch (Figure 7A), and the other pSIB527 carried the *gfp* gene that was regulated with the Tet-Off switch (Figure 7B). Both of them can deliver the relevant transcription unit and its transactivator into yeast cells in a single step. When a strain CaTe-Off in which both systems were integrated was grown lacking both camphor and doxycyclin (Dox), significant expression of both fluorescent proteins was observed (Figure 7C). Moreover, the addition of either drug resulted in drastic decrease of a corresponding reporter protein, whereas the other type of fluorescence was detected robustly (Figure 7, D and E). In accordance with those data, expression of both reporters was repressed when both camphor and Dox were supplied in the medium (Figure 7F). These results indicated that it was possible to regulate genes separately with the two systems in a single strain.

### Perspectives

There are increasing needs for effective switches in biotechnology-related fields, and the present study describes a Camphor-Off system that can realize tight regulation of transcription in yeast with camphor, an inexpensive waste product of the Kraft pulping process. This switch turns-off the expression of genes of interest only by supplementing camphor in a micromolar-order concentration that has little detectable effect on yeast physiology, distinguishing it from most of other repressive switches previously developed, such as the *MET3* and *PHO5* promoters. In other words, this new system can be useful in many contexts, not only in laboratories but also in industry, where kiloliter to megaliter scale fermentation may be performed. It is noteworthy that the Camphor-Off switch can regulate expression in 1 megaliter of yeast at a cost of US$160 as of 2014. From this point of view, the Camphor-Off switch is expected to be one of the most valuable switches for regulating gene expression.

## 
